# Emotional Intelligence, Psychological Well-Being and Burnout of Active and In-Training Teachers

**DOI:** 10.3390/ijerph19063514

**Published:** 2022-03-16

**Authors:** Susana Lucas-Mangas, Lorena Valdivieso-León, Ivette Margarita Espinoza-Díaz, Jordi Tous-Pallarés

**Affiliations:** 1 Department of Psychology, University of Valladolid, 47002 Valladolid, Spain; susana.lucas@uva.es; 2 Department of Psychology, University of Rovira i Virgilli, 43007 Tarragona, Spain; ivettemargarita.espinoza@urv.cat (I.M.E.-D.); jordi.tous@urv.cat (J.T.-P.)

**Keywords:** burnout, emotional intelligence, emotional regulation, psychological well-being

## Abstract

The main activating variables of psychological well-being and Emotional Intelligence that influence teachers include the process of evaluating well-being, their motivation, and their ability to perceive and regulate sources of stress and burnout. The relationship and influence of psychological well-being and emotional intelligence was analyzed with the adequate regulation of burnout. Those who participated included 386 active teachers (55%), and teachers in training (45%), studying for degrees in Pre-School and Primary Education, and Master’s degrees in Secondary Education Teacher Training of which 71.5% were women. The following were used: Psychological Well-Being Scales, Trait Meta-Mood Scale and the Spanish Burnout Inventory. Pearson’s correlation analysis and multiple regression analysis were performed. The results showed that enthusiasm for the teaching job is related to psychological well-being, especially domain of the environment and personal growth. Multiple regression analysis made it possible to establish a predictive model of well-being, showing that psychological well-being is the main adjustment predictor and/or the mismatch in the work of the teaching staff in both samples, through an adequate regulation of positive relationships, mastery of their environment and having a purpose in life.

## 1. Introduction

In recent years, many studies have appeared that attempt to identify the influence of teachers’ work on their mental health and well-being [1,2]. Psychological well-being depends on interpersonal relationships, satisfaction, and professional success as a teacher [3,4], and is associated with happiness, satisfaction, subjective well-being, or quality of life, as perceived by teachers at the individual level [5,6,7], as well as emotional intelligence [8,9].

Among the main activating variables of psychological well-being that influence teachers are the evaluation of experienced well-being evaluation, their motivation, and their ability to perceive and regulate sources of stress and burnout at work [10,11,12]. Therefore, emotional intelligence could be an activating coping strategy that provides information on the well-being of teaching staff, that is, on how emotions are perceived, assimilated, understood, and regulated in their work environment, as this is a key element of emotional self-regulation that the teacher will use to manage stress and burnout situations [13].

In the past decades, psychological well-being and emotional intelligence have been providing an increasing motivation to lay the foundations of a solid theoretical base that would serve as a reference in the construction of instruments such as the empirical works carried out with the Trait Meta-Mood Scale (TMMS) [14,15,16], in attempts to identify whether this concept explains burnout variables, as shown in the studies of Pedrosa et al. [17], Espinoza, Tous and Vigil [18] y de Merino et al. [19], in its link with life satisfaction and psychological well-being [20,21]. Various components of quality of life are also studied, such as physical and mental health [22,23,24,25,26,27], or their positive impact on interpersonal and cooperative group skills.

However, the continued presence of stressful conditions in teachers’ work, can trigger situations of emotional discomfort and dissatisfaction, and lead the teacher to experience burnout [28,29]. Therefore it becomes even more relevant to outline the challenge of delving into an integrative model derived from neuroscience that, when applied to the educational field, contributes to a greater understanding of how cognitive and emotional processes are integrated, so that emotions become vital elements of the human being in their relationship with the environment [30].

Interest is mainly focused on the adoption of a preventive role, and the promotion of quality of life in the professional performance of teachers, related to the training of instructors, to model their work environments [31,32,33,34,35].

Therefore, the intention is to evaluate the relationship of the factors of emotional intelligence and psychological well-being with the burnout of two groups of teachers (one active and the other in training, in Pre-School, Primary and Secondary education) in order to analyze to what extent these variables allow for the establishment of a predictive model of psychological well-being through multiple regression analysis. It is considered that it is possible that the factors of psychological well-being and emotional intelligence have a special influence as a strategy to minimize burnout effects, as well as providing a perception of greater well-being in teachers [34,36,37,38].

## 2. Materials and Methods

### 2.1. Participants

A total of *n* = 386 teachers participated (71.5% women; 28.5% men); the age mean was 31.4 with an SD = 10.9, where the minimum was 18 and the maximum, 64. Out of all participants, 45% were studying for a degree in Pre-School and Primary Education and a Master’s degree in Secondary Education Teacher Training, and 55% taught these degrees. The professional teachers worked in centers in the province of Tarragona (Spain) and the students studied at Rovira i Virgili University. Teachers with more than 10 years of professional experience represented 36% of the total, while the rest of those surveyed had less experience.

### 2.2. Instruments

The Spanish adaptation of Ryff’s Psychological Well-Being Scales (EBP) [39] is a reduced version of 29 items that are adapted from the developed questionnaire. It is a test to measure psychological well-being through six dimensions: self-acceptance (α = 0.84), or the intention to feel good about oneself; the ability to maintain positive relationships (α = 0.78) with other people; autonomy (α = 0.70) to resist situations of social pressure; domain of the environment (α = 0.82), to choose or create favorable environments; setting goals or purpose in life (α = 0.70) to provide some meaning to life, and finally; valuing their life as a situation of personal growth (α = 0.71).

The TMMS-24 test, the Spanish validation of the Trait Meta-Mood Scale [40] in its original version [16] is used. This is a scale of 24 items, which contains three key dimensions of emotional intelligence, with 8 items in each. It evaluates Emotional Attention (α = 0.90) or emotional perception, understood as the individuals’ ability to perceive the level of attention they pay to their emotions; Emotional Clarity (α = 0.90), or understanding feelings, referring to the ability of an individual to clearly identify the type of emotion they experience; and Emotional Repair (α = 0.86), or emotional regulation, measuring the ability of a person to correctly regulate their emotional states.

The Spanish Burnout Inventory (SBI) [29] is a 25-item questionnaire, answered by the participants using a 5-point Likert scale. This test assesses 4 factors and a general scale that allows for a diagnosis of enthusiasm for work (α = 0.90), mental exhaustion (α = 0.85), indolence (α = 0.74), guilt (α = 0.82), and a general burnout scale with a reliability of α = 0.85.

### 2.3. Procedures

The test was administered through a web platform hosted on our laboratory servers. The participants were contacted through companies and institutions that collaborate with our team on other research and transfer projects. They were asked to spread the link through which they could access the platform hosting the test, and where they could leave their answers.

The platform had a filter that guaranteed that the participants voluntarily agreed to be part of the study, without any type of coercion or financial remuneration, and particpants also stated that they had been informed that the administration of the scale was completely anonymous and governed by the Data Protection Act.

### 2.4. Data Analysis

A Pearson correlation analysis was carried out to identify the relationship between the emotional intelligence and psychological well-being of the total sample, which included active teachers and teachers-in-training in Pre-School, Primary and Secondary Education.

Subsequently, the transformation of Fisher’s Z was carried out to determine the confidence intervals in the comparison of the Pearson correlation coefficients in the two groups of teachers (one active and the other in training in Pre-School, Primary and Secondary Education) in order to identify significant differences.

Finally, in order to determine the variables that influenced the total sample of active teachers and those in training, a multiple regression analysis was carried out using successive steps, looking at the sample of only active teachers and that of only teachers-in-training separately. The goal was to identify the factors that explain the regulation of burnout levels in each case.

## 3. Results

In the Pearson correlation analysis, we found that both emotional intelligence and psychological well-being favor enthusiasm for work and decrease the levels of depersonalization, indolence, and cynicism, in addition to significantly reducing the levels of burnout. It should also be noted that the relationship between the psychological well-being scales was especially high and consistently significant in the regulation of this well-being (see Table 1).

Regarding the teaching staff in training, Table 2 shows that the only emotional intelligence variable that is related to the regulation of burnout is emotional repair. However, the relationship shown between all scales of psychological well-being during their correct regulation is also noteworthy.

Likewise, in Table 3, we see that, in the case of active teachers, the relationship between emotional intelligence and psychological well-being and burnout is characterized by the following findings: regarding emotional intelligence, active teachers face burnout through emotional clarity, the same way as teachers-in-training. Regarding emotional repair, the relationship that all psychological well-being scales have with burnout is noteworthy, with the domain of the environment being especially remarkable (*r* = −0.540; *p* < 0.01), personal growth (*r* = −0.492; *p* < 0.01), positive relationships (*r* = −0.479; *p* < 0.01), self-acceptance (*r* = −0.450; *p* < 0.01) and, to a lesser extent, but not less important, those of purpose in life (*r* = −0.362; *p* < 0.01) and autonomy (*r* = −0.279; *p* < 0.01).

Once the correlation analyses were carried out for both samples (active teachers and teachers in training), and as can be seen in Table 4, the result of Fisher’s Z analysis only showed two significant differences in relation to enthusiasm for work with the scales of psychological well-being–domain of the environment (E r = 0.258, *p* < 0.01; P r = 0.437, *p* < 0.01), and with personal growth (E r = 0.305, *p* < 0.01; P r = 0.473, *p* < 0.01). These data clearly reflect that enthusiasm for work, although positive, is also slightly lower in teachers-in-training than in active teachers, which could be linked to the fact that when academic preparation is part of professional practice, then the domain of the environment capacity increases, and so does personal growth, thus favoring the increase in enthusiasm for work.

Regarding the multiple regression analysis, we found that, in the total sample of teachers-in-training and active teachers, the variables that explain well-being and/or discomfort derived from burnout, are positive relationships as follows: domain of the environment and purpose in life. These factors explain 28.2% of the variance in burnout. In the case of the sample of teachers in training, 23.7% of the variance is explained by positive relationships and mastery of the environment. Finally, in the case of the sample of active teachers, we found that, as in the total sample, positive relationships, domain of the environment, and purpose in life explained 35.7% of the total variance in burnout. It is worth mentioning that none of the emotional intelligence scales became a part of the predictive model in any of the cases. Therefore, as can be seen in Table 5, we can affirm that an adequate adjustment of active/in-training teachers with their environment, would imply having the ability to interact in a positive way, to be in control of their environment, and have an adequate definition of their purpose in life, which makes it clear that psychological well-being is the main predictor of adjustment and/or mismatch between active and in-training teachers facing burnout.

## 4. Discussion

The objective of the present work was to analyze the relationship between emotional intelligence and psychological well-being and burnout in active and in-training teachers. Another objective was to identify the factors that had the greatest influence in each of the sub-samples, in order to identify significant differences between teachers-in-training and active teachers.

As can be seen in the results of the study, the analysis of the total sample highlights the correlations between emotional intelligence and psychological well-being with total burnout in each of its variables (Table 1), in which it is observed that the relationship between the variables of burnout and total burnout has higher correlations when associated with psychological well-being than when associated with emotional intelligence, especially with the scales of positive relationships, domain of the environment and personal growth. Regarding emotional intelligence, the correlation between the total burnout scale with emotional repair was the most representative.

When analyzing the correlations between the same scales in the sample of teachers in training (Table 2), high correlations were observed in the emotional repair variables of the emotional intelligence scale, while, in the total burnout scale, all were significant, but those of most importance were positive relationships, domain of the environment and personal growth. In relation to the sample of active teachers, the highest correlations between burnout and psychological well-being were observed in positive relationships, domain of the environment, personal growth, self-acceptance, and purpose in life (Table 3).

In relation to the comparative analysis between both sub-samples (Table 4), it can be seen that in the case of emotional intelligence, the emotional clarity scale is significant, and is therefore important in the professional development of active teachers; however, in the case of teachers-in-training, it was not significant. Regarding psychological well-being, it can be observed that the correlations of all its scales are significant and contribute to a greater extent to the decrease in total burnout in both subsamples and that, likewise, psychological well-being contributes to the decrease in total burnout in active teachers and teachers-in-training, which is ratified in the sample of active teachers (Table 4).

Once the correlations between both sub-samples were analyzed, using the results in Table 4, the analysis of the differences between the correlations of both sub-samples was carried out using Fisher’s Z in order to find significant differences between the correlations in each one of the scales of burnout and total burnout. In this sense, the analysis was able to confirm two significant differences between both samples (active teachers and teachers-in-training): enthusiasm for work, which is more related to psychological well-being, especially with the domain of the environment and personal growth, whose difference in both cases was statistically significant, and consistent with enthusiasm for work. In this sense, professional success and interpersonal relationships are linked to the psychological well-being of the teaching staff [1,3,4].

Regarding the multiple regression analysis, it revealed that, when introducing the psychological well-being and emotional intelligence scales, it only introduced the psychological well-being scales, thus ruling out the emotional intelligence scales as an influencing factor. In this sense, the result of the analysis allowed us to establish a predictive model of the psychological adjustment that active and in-training teachers make against burnout, thus making it clear that psychological well-being is the main predictor of adjustment and/or maladjustment to the work of the active teacher and teachers-in-training. In this sense, the analysis presented in Table 5 shows us the percentage of variance explained in the total sample, in the sub-sample of teachers-in-training and active teachers.

Regarding the total sample, it is observed that the model explains 28.2% of the total variance in burnout regulation with positive relationships (6.3%), domain of the environment (20.6%), and purpose in life (1.3%). In the sub-sample of teachers in training, the model revealed that positive relationships (18.9%) and domain of the environment (4.8%) explain 23.7% of the variance in the environment. Finally, in the case of active teaching staff, positive relationships (4.6%), domain of the environment (29.2%), and purpose in life (14.9%) were the variables that explained 35% of the variance in psychological well-being in this sub-sample.

As can be seen, positive relationships, domain of the environment and purpose in life were the three scales that were constantly part of the predictive model of psychological well-being in the total sample, in teachers who are in training and those in active service. However, it is important to clarify that the scale of purpose in life did not become part of the model in the sample of teachers in training; therefore, it is considered interesting that, within the training lines of these students and future teachers, the importance of having a clear purpose in life is reinforced (from a preventive approach to quality of life).

Consequently, these results reflect their possible usefulness for the design of an educational model that promotes adequate teacher training, which has repercussions for the quality of education, psychological well-being, and future development of a teacher’s professional career, in accordance with the Objective of Sustainable Development 4.7. In this sense, various authors propose the study of the steps that should be followed in self-regulated learning, and the relevance of goal-setting, in the development process of each educational project [41], and in the development of socially responsible entrepreneurial projects with the community [42].

### Limitations

Because all data were collected concurrently, temporal precedence could not be established.

It is suggested that future research is carried out in which the study is extended to other professional profiles of active and in-training teachers.

Likewise, it is recommended to study the mediating role of other sociodemographic variables, such as age and the influence of cultural differences [31], as well as the intervention of variables such as stress-management strategies when teachers have to face difficulties [43], or self-efficacy in the development of a socially responsible career project while promoting quality of life [42].

## 5. Conclusions

The results obtained from this research contribute to a collaboration with the 2030 Agenda [44], as a work plan for the coming years in favor of people, the planet and prosperity, in order to contribute to the promotion of a society whose development model is based on sustainability and resilience, with 17 objectives and 169 goals. The universal characteristics of transformation and inclusion that these objectives and goals represent are the greatest challenges that humanity has ever set itself. The SDGs that are being developed during this research are mainly SDG3: Good Health and Well-Being, and SDG4: Education of Quality.

Within the educational community, the well-being of teachers is key to achieving quality education [45], leading to a higher performance [26], and the identification of the relevance of these variables means an even greater impact on the development and evaluation of programs when dealing specifically with teachers in training to become future childhood and youth educators [35].

Likewise, UNESCO [46] affirms that education for sustainable development (ESD), based on target 4.7 of the SDGs, and as a facilitating element of the 17 SDGs, is the basis of the necessary transformation: jointly emphasizing cognitive skills, social and emotional learning, and action skills, for the individual and social dimensions of this transformation.

According to these references, in order to develop skills that facilitate participation at a local and global level, it is necessary, first of all, to become aware of the transcendence of the socio-affective dimension of education. That is why the results obtained in this study regarding psychological well-being and the psychosocial climate of teachers will contribute to providing teachers with tools that empower them in their work as educators: working on emotional self-awareness and its relationship with citizen participation; active listening and empathy, the key to communication in a globalized world; the management of frustration and realistic and positive self-knowledge in the exercise of citizenship; cooperation in conflict resolution; and the group as a space for affective growth, training and social participation, in order to generate a healthy psychosocial climate (the group as a space for affective growth, and model of teacher support in Transformative Education; individual roles and constructive management of conflicts in the group to build global citizenship) [47].

As a future line of work, we intended to study howthese factors can influence the analysis of psychological well-being, emotional intelligence, and burnout from a multicultural perspective and when focusing on gender.

## Figures and Tables

**Table 1:** Relationship between emotional intelligence and psychological well-being and burnout in active teachers and teachers-in-training.

	IT	DP	IN	C	Burnout Total
Emotional Intelligence					
Emotional Attention	**0.133** **	0.063	0.078	0.080	0.015
Emotional Clarity	**0.168** **	**−0.165** **	−0.067	**−0.164** **	**−0.178** **
Emotional Repair	**0.192** **	**−0.210** **	**−0.208** **	**−0.189** **	**−0.268** **
Psychological Well-Being					
Self-Acceptance	**0.302** **	**−0.259** **	**−0.265** **	**−0.248** **	**−0.359** **
Positive Relationships	**0.390** **	**−0.348** **	**−0.301** **	**−0.240** **	**−0.454** **
Autonomy	0.110 *	**−0.230** **	**−0.233** **	**−0.252** **	**−0.258** **
Domain of the Environment	**0.333** **	**−0.330** **	**−0.377** **	**−0.315** **	**−0.454** **
Purpose in Life	**0.347** **	**−0.179** **	**−0.256** **	**−0.205** **	**−0.330** **
Personal Growth	**0.374** **	**−0.275** **	**−0.292** **	**−0.256** **	**−0.406** **

Note. ** *p* < 0.01; * *p* < 0.05; IT = Enthusiasm for Work; DP = Depersonalization; IN = Indolence; C = Guilt.

**Table 2:** Relationship between emotional intelligence and psychological well-being and burnout in teachers-in-training.

	IT	DP	IN	C	Burnout Total
Emotional Intelligence					
Emotional Attention	0.066	0.142	0.090	0.138	0.090
Emotional Clarity	0.104	−0.122	0.003	−0.113	−0.099
Emotional Repair	**0.199** **	**−0.264** **	**−0.223** **	**−0.198** **	**−0.309** **
Psychological Well-being					
Self-acceptance	**0.262** **	**−0.228** **	**−0.203** **	−0.183 *	**−0.304** **
Positive relationships	**0.355** **	**−0.331** **	**−0.298** **	−0.165 *	**−0.435** **
Autonomy	0.112	**−0.214** **	**−0.213** **	**−0.208** **	**−0.247** **
Domain of the environment	**0.258** **	**−0.314** **	**−0.341** **	**−0.265** **	**−0.410** **
Purpose in life	**0.350** **	−0.155 *	**−0.214** **	−0.156 *	**−0.304** **
Personal growth	**0.305** **	**−0.231** **	**−0.269** **	**−0.209** **	**−0.352** **

Note: ** *p* < 0.01; * *p* < 0.05; IT = Enthusiasm for Work; DP = Depersonalization; IN = Indolence; C = Guilt.

**Table 3:** Relationship of emotional intelligence and psychological well-being and burnout in active teachers.

	IT	DP	IN	C	Burnout Total
Emotional Intelligence					
Emotional Attention	0.153 *	0.043	0.072	0.003	−0.008
Emotional Clarity	**0.237** **	**−0.217** **	−0.135 *	**−0.207** **	**−0.260** **
Emotional Repair	**0.227** **	**−0.208** **	**−0.199** **	−0.163 *	**−0.275** **
Psychological Well-being					
Self-acceptance	**0.381** **	**−0.329** **	**−0.332** **	**−0.303** **	**−0.450** **
Positive relationships	**0.425** **	**−0.370** **	**−0.304** **	**−0.311** **	**−0.479** **
Autonomy	0.117	**−0.256** **	**−0.252** **	**−0.298** **	**−0.279** **
Domain of the environment	**0.437** **	**−0.395** **	**−0.417** **	**−0.344** **	**−0.540** **
Purpose in life	**0.359** **	**−0.205** **	**−0.297** **	**−0.261** **	**−0.362** **
Personal Growth	**0.473** **	**−0.350** **	**−0.319** **	**−0.289** **	**−0.492** **

Note: ** *p* < 0.01; * *p* < 0.05; IT = Enthusiasm for Work; DP = Depersonalization; IN = Indolence; C = Guilt.

**Table 4:** Significant differences in the relationship between emotional intelligence and psychological well-being and burnout in active and in-training teachers.

	IT		DP		IN		C		Burnout Total	
	E	P	E	P	E	P	E	P	E	P
Emotional Intelligence										
Emotional Attention	0.066	0.153 *	0.142	0.043	0.090	0.072	0.138	0.003	0.090	−0.008
Emotional Clarity	0.104	**0.237** **	−0.122	**−0.217** **	0.003	−0.135 *	−0.113	**−0.207** **	−0.099	**−0.260** **
Emotional Repair	**0.199** **	**0.227** **	**−0.264** **	**−0.208** **	**−0.223** **	**−0.199** **	**−0.198** **	−0.163 *	**−0.309** **	**−0.275** **
Psychological Well-Being										
Self-Acceptance	**0.262** **	**0.381** **	**−0.228** **	**−0.329** **	**−0.203** **	**−0.332** **	−0.183 *	**−0.303** **	**−0.304** **	**−0.450** **
Positive Relationships	**0.355** **	**0.425** **	**−0.331** **	**−0.370** **	**−0.298** **	**−0.304** **	−0.165 *	**−0.311** **	**−0.435** **	**−0.479** **
Autonomy	0.112	0.117	**−0.214** **	**−0.256** **	**−0.213** **	**−0.252** **	**−0.208** **	**−0.298** **	**−0.247** **	**−0.279** **
Domain of the Environment	**0.258** **	**0.437** **	**−0.314** **	**−0.395** **	**−0.341** **	**−0.417** **	**−0.265** **	**−0.344** **	**−0.410** **	**−0.540** **
Purpose in Life	**0.350** **	**0.359** **	−0.155 *	**−0.205** **	**−0.214** **	**−0.297** **	−0.156 *	**−0.261** **	**−0.304** **	**−0.362** **
Personal Growth	**0.305** **	**0.473** **	**−0.231** **	**−0.350** **	**−0.269** **	**−0.319** **	**−0.209** **	**−0.289** **	**−0.352** **	**−0.492** **

Note: ** *p* < 0.01; * *p* < 0.05; IT = enthusiasm for work; DP = depersonalization; IN = indolence; C = guilt; E = teachers in training; *p* = active teachers; underlined values: the differences between all relationships f were analyzed and statistically significant differences were underlined.

**Table 5:** Psychological well-being and emotional intelligence variables that influence the regulation of burnout in active teachers and teachers in training.

Variables That the Model Introduces	TS		TT		AT	
	R^2^	β	R^2^	β	R^2^	β
Self-Acceptance	-	-	-	-	-	-
Positive Relationships	0.063 ***	−0.297 ***	0.189 ***	−0.435 ***	0.046 ***	−0.259 ***
Autonomy	-	-	-	-	-	-
Domain of the Environment	0.206 ***	−0.454 ***	0.048 ***	−0.255 ***	0.292 ***	−0.540 ***
Purpose in Life	0.013 **	−0.271 **	-	-	0.019 **	−0.149 **
Personal Growth	-	-	-	-	-	-
Explained Total Variance	**28.20%**		**23.70%**		**35.70%**	

Note. *** *p* < 0.001; ** *p* < 0.01; TT = teachers in training; AT = active teachers; TS = total sample; β = coefficient.

## Data Availability

The data presented in this study are available on reasonable request from the corresponding author.
